# Telomeric injury by KML001 in human T cells induces mitochondrial dysfunction through the p53-PGC-1α pathway

**DOI:** 10.1038/s41419-020-03238-7

**Published:** 2020-12-02

**Authors:** Madison Schank, Juan Zhao, Ling Wang, Zhengke Li, Dechao Cao, Lam Nhat Nguyen, Xindi Dang, Sushant Khanal, Lam Ngoc Thao Nguyen, Bal Krishna Chand Thakuri, Stella C. Ogbu, Zeyuan Lu, Jinyu Zhang, Xiao Y. Wu, Zheng D. Morrison, Mohamed El Gazzar, Shunbin Ning, Jonathan P. Moorman, Zhi Q. Yao

**Affiliations:** 1 Center of Excellence in Inflammation, Infectious Disease and Immunity, James H. Quillen College of Medicine, East Tennessee State University, Johnson City, TN 37614 USA; 2 Department of Internal Medicine, Division of Infectious, Inflammatory and Immunologic Diseases, Quillen College of Medicine, ETSU, Johnson City, TN 37614 USA; 3 Hepatitis (HCV/HBV/HIV) Program, James H. Quillen VA Medical Center, Department of Veterans Affairs, Johnson City, TN 37614 USA

**Keywords:** Immunology, Diseases

## Abstract

Telomere erosion and mitochondrial dysfunction are prominent features of aging cells with progressive declines of cellular functions. Whether telomere injury induces mitochondrial dysfunction in human T lymphocytes, the major component of adaptive host immunity against infection and malignancy, remains unclear. We have recently shown that disruption of telomere integrity by KML001, a telomere-targeting drug, induces T cell senescence and apoptosis via the telomeric DNA damage response (DDR). In this study, we used KML001 to further investigate the role and mechanism of telomere injury in mitochondrial dysregulation in aging T cells. We demonstrate that targeting telomeres by KML001 induces mitochondrial dysfunction, as evidenced by increased mitochondrial swelling and decreased mitochondrial membrane potential, oxidative phosphorylation, mitochondrial DNA content, mitochondrial respiration, oxygen consumption, glycolysis, and ATP energy production. Mechanistically, we found that the KML001-induced telomeric DDR activated p53 signaling, which in turn repressed the expression of peroxisome proliferator-activated receptor-gamma coactivator 1 alpha (PGC-1α) and nuclear respiratory factor 1 (NRF-1), leading to T cell mitochondrial dysfunction. These results, forging a direct link between telomeric and mitochondrial biology, shed new light on the human T cell aging network, and demonstrate that the p53-PGC-1α-NRF-1 axis contributes to mitochondrial dysfunction in the setting of telomeric DDR. This study suggests that targeting this axis may offer an alternative, novel approach to prevent telomere damage-mediated mitochondrial and T cell dysfunctions to combat a wide range of immune aging-associated human diseases.

## Introduction

Societies around the world are experiencing a dramatic increase in elderly populations. This rise in the aging population is accompanied by a sharp increase in the prevalence of age-related conditions and diseases, ranging from increased susceptibility to infections to reduced responses to vaccines, increased incidence of cancers, cardiomyopathy, metabolic disorders (diabetes), muscle atrophy, and neurodegenerative disorders (Alzheimer’s), all of which contribute to comorbidity and mortality in the elderly^1–3^. Compelling evidence from molecular and cellular analyses in both humans and animals has highlighted the importance of shortened telomeres and impaired mitochondria, hallmarks of cell senescence—a quiescent, non-replicative, and functionally anergic state, in the age-related decline of cellular functions in the elderly^4–6^. Telomere erosion and mitochondrial dysfunction are prominent characteristics of aging cells in high-turnover tissues or cells such as T lymphocytes. Indeed, the progressive loss of T cell proliferative capacity and/or responsiveness to antigenic challenges during aging directly correlate with a gradual shortening of telomeres and a decline in mitochondrial functions^7,8^.

Telomeres are hexameric DNA repeats (TTAGGG) at the ends of the chromosomes in association with a complex of shelterin proteins, including telomere repeat binding factor 2 (TRF2)^9^. Telomere integrity is a key feature of linear chromosomes that preserves genome stability. Conversely, telomere erosion via the DNA damage response (DDR) is a hallmark of cell senescence that drives cell dysfunction or apoptosis^10–12^. Therefore, telomere length has been deemed a biological clock controlling cell survival and function, whereas telomere erosion has been considered a faithful readout of cell aging^7,12^. Previously, we and others have shown that chronic viral (HCV, HIV) infection can drive premature T cell aging, as evidenced by accelerated telomere erosion, leading to T cell phenotypes and functions similar to those observed in the elderly^13–18^. Also, we have recently shown that disruption of telomere integrity by KML001 (sodium meta-arsenite, NaAsO_2_, a telomere-targeting drug)^19,20^ induces T cell senescence and apoptosis^21^. How telomere erosion specifically leads to T cell aging and dysfunction, however, remains unclear.

Aged cells show decreased mitochondrial DNA content and increased reactive oxygen species (ROS), causing a deficiency in cell respiration, glycolysis, and ATP production, all of which underlie the gradual loss of vigor in the elderly^22,23^. Mitochondrial compromise in cell aging is evidently reinforced by recent studies revealing the repression of master regulators of mitochondrial biogenesis and metabolism, peroxisome proliferator-activated receptor-gamma coactivator 1 alpha and beta (PGC-1α and PGC-1β), across aging tissues in mice^24,25^. Notably, PGC-1α controls several critical transcription factors, including nuclear respiratory factor 1 (NRF-1) and estrogen-related receptor alpha (ERRα), which activate the expression of key metabolic genes regulating cellular growth and nuclear genes required for respiration, heme-biosynthesis, and mitochondrial DNA transcription and replication^26–28^. The role and mechanisms of PGC-1α/1β, NRF-1, and ERRα in human aging, especially in T lymphocyte and immune aging, remain largely unknown.

T cells play a crucial role in defending the host against infections and malignancies; however, the mechanisms affecting their senescence remain incompletely understood. Of note, while telomeric DDR and mitochondrial dysfunction have been intensively investigated in different settings, these studies often investigate these two events separately. In this study, we employed human CD4 T cells treated with KML001 as a model system to investigate an intimate link between telomere damage and mitochondrial dysfunction during human T cell aging. We provide solid evidence that targeting telomeres by KML001 triggers telomeric DDR via disruption of DNA repair machineries, such as the base excision repair (BER) pathway, and induces mitochondrial dysfunction in T cells via the p53-PGC-1α-NRF-1 axis. These studies shed new light on the T cell aging network and offer an alternative, novel approach for preventing telomere damage-mediated mitochondrial dysfunction to combat a wide range of immune aging-associated human diseases.

## Materials and methods

### Subjects

The study protocol was approved by the institutional review board (IRB) of East Tennessee State University and James H. Quillen VA Medical Center (ETSU/VA IRB, Johnson City, TN). The study subjects were composed of two populations: 55 healthy subjects (HS), obtained from the BioIVT (Gray, TN) used for KML001 treatment and p53/TRF2 manipulation experiments, and 16 HS compared to 32 age-matched people living with HIV (PLHIV) on ART with undetectable viremia (HIV-RNA<20). All HS were negative for HBV, HCV, and HIV infections. All HIV participants were adults and signed an informed consent form.

### Cell isolation and culture

PBMCs (peripheral blood mononuclear cells) were isolated from whole blood of HS or PLHIV by Ficoll density centrifugation (GE Healthcare, Piscataway, NJ). CD4 T cells were purified from PBMCs using the CD4^+^ T Cell Isolation Kit and a MidiMACS™ Separator (Miltenyi Biotec, Auburn, CA). The CD4 T cells were cultured in RPMI-1640 medium containing 10% fetal bovine serum (FBS) (Atlanta Biologicals, Flowery Branch, GA), 100 IU/ml penicillin, and 2 mM L-glutamine (Thermo Scientific, Logan, Utah), and treated with 5 or 10 µM KML001 (Sigma-Aldrich, St. Louis, MO) or DPBS control for different times as indicated and maintained at 37 °C in a 5% CO_2_ incubator.

### Flow cytometry analysis

MitoTracker Green (MG) and MitoTracker Orange (MO) were obtained from Invitrogen (Carlsbad, CA; Cat #M-7514 and M-7511) and used according to the manufacturer’s protocol. The MG and MO mitochondrion-selective probes allow for the quantification of mitochondrial mass and oxidative phosphorylation (OXPHOS), respectively. Purified CD4 T cells were cultured with 100 nM MG or 500 nM MO for 30 min at 37 °C and then analyzed by flow cytometry. For analysis of apoptosis and cellular ROS, cells were stained with Annexin V-PE using the BD Pharmingen PE Annexin V Apoptosis Detection Kit I (BD Biosciences, San Jose, CA) and CellROX Green ROS Detection kit (Thermo Fisher Scientific, Waltham, MA) according to manufacturer’s protocol. For analysis of mitochondrial ROS, cells were stained with 5 μM MitoSOX Red mitochondrial superoxide indicator (Invitrogen) at 37 °C for 30 min and then analyzed by flow cytometry.

To analyze cycling, non-cycling, naive, and memory CD4 T cells by flow cytometry, the following fluorochrome-conjugated antibodies were used: PE anti-human CD4 (Cat #300508), PerCP anti-human CD45RA (Cat# 304156), Alexa Fluor 647 anti-human CD71 (Cat #334118) (all from BioLegend), Alexa Fluor 488 PGC1α (Novus Biologicals, Littleton, CO; Cat #NBP1-04676AF488;), and Alexa Fluor 488 Anti-NRF-1 (Abcam, Cambridge, MA; Cat #ab200976). For HS vs PLHIV, PBMCs were isolated and analyzed by flow cytometry. For KML001 treatment, CD4 T cells were isolated from PBMCs and cultured in the presence of KML001 or DPBS control for 48 h and thus were not stained with CD4-PE. The cells were stained with anti-CD45RA, anti-CD71, and anti-CD4 (for PBMCs) antibodies, followed by incubation with fixation/permeabilization with Foxp3 transcription factor staining buffer (Invitrogen; Cat #00-5523-00). Intracellular staining was then performed with anti-PGC1α and anti-NRF-1 antibodies. Unstained, isotype, and single positive controls were used for gating and compensation. The cells were assayed using a BD Accuri C6 Plus flow cytometer and the data were analyzed by FlowJo v10.0 software.

### ATP Luminescence

Purified CD4 T cells were plated in a 96-well plate. A standard curve was generated by preparing 8000 nM ATP (Sigma-Aldrich, Cat #A7699) in 10% FBS RPMI-1640 culture medium and serial (1:1 ratio) dilution of ATP with medium. Approximately 100 µl of CellTiter-Glo Reagent (Promega, Madison, WI; Cat# G7570) was added (1:1 ratio) in all wells. Luminescence was measured using the Synergy H1 BioTek plate reader after mixing for 2 min (to induce cell lysis) and holding 10 min (to stabilize the luminescent signal).

### Seahorse XFp Cell Mito Stress Test

Seahorse XFp Cell Mito Stress Tests (Agilent Technologies, Santa Clara, CA) were performed according to the manufacturer’s protocol using an XFp instrument. Two days prior to the assay, CD4 T cells were isolated and cultured in 10% FBS RPMI-1640 medium with 5 µM KML001 or DPBS control. One day prior to the assay, Seahorse mini-cartridges were hydrated in a non-CO_2_ incubator overnight. On the day of the assay, seahorse mini-plates were coated with 25 μl of 0.1 mg/ml poly-D-lysine (Thermo Fisher Scientific; Cat # A3890401) for 1 h at room temperature. The cells were harvested and washed by DPBS once. Approximately 200,000 cells per well were plated in pre-coated mini plates and cultured in Seahorse XF RPMI assay medium supplemented with 1 mM glucose, 100 µM pyruvate, and 1 mM glutamine. Data analysis was performed using the Seahorse Wave software, including the Seahorse Mito Stress Test report generator.

### Gene microarray

CD4 T cells were isolated from HS and cultured with 5 μM KML001 or DPBS control for 48 h. Approximately 6 × 10^6^ cells per group were sent to Arraystar Inc. (Rockville, MD) for gene expression analysis. The heatmap was generated using an online heatmapper software^29^.

### Real-time qPCR and reverse transcription-quantitative PCR (RT-qPCR)

To measure mitochondrial DNA (mtDNA) and nuclear DNA (nuDNA) content, genomic DNA was extracted from CD4 T cells using the PureLink genomic DNA kit (Thermo Fisher Scientific). DNA concentration was measured by the Synergy H1 BioTek plate reader. The primers used for mitochondrial and nuclear DNA PCR are shown in Table 1. Approximately 25 ng of genomic DNA was used in PCR reactions. The PCR cycling conditions were: 1 cycle at 50 °C for 2 min, 1 cycle at 95 °C for 10 min, and 40 cycles at 95 °C for 15 s and 62 °C for 60 s. The averages of mtDNA and nucDNA Cq values from triplicate reactions were calculated. The mitochondrial DNA content was determined using the following equations: a. ΔCq = (nucDNA Cq – mtDNA Cq). b. Relative mitochondrial DNA content = 2 × 2^ΔCq^ ^30^.

**Table 1:** PCR Primers.

Gene	Forward primer	Reverse primer
PPARGC1A	5′-GGCAGAAGGCAATTGAAGAG-3′	5′-TCAAAACGGTCCCTCAGTTC-3′
PPARGC1B	5′-CCAGATACACTGACTACGATTCCAA-3′	5′-GGGTTAAGGCTGTTATCAATGCA-3′
ESSRA	5′-GGCCCTTGCCAATTCAGA-3′	5′-GGCCTCGTGCAGAGCTTCT-3′
NRF-1	5′-GATGCTTCAGAATTGCCAACC-3′	5′-CTCATCTCACCTCCCTGTAAC-3′
TFAM	5′-CCGAGGTGGTTTTCATCTGT-3′	5′-GCATCTGGGTTCTGAGCTTT-3′
PPARA	5′-GGCGAGGATAGTTCTGGAAGC-3′	5′-CACAGGATAAGTCACCGAGGAG-3′
SOD1	5′-GTGCAGGTCCTCACTTTAAT-3′	5′-CTTTGTCAGCAGTCACATTG-3′
GPX1	5′-TTCCCGTGCAACCAGTTTG-3′	5′-TTCACCTCGCACTTCTCGAA-3′
PCK1	5′-CTGTGACGGCTCTGAGGAGGAGAA-3′	5′-CCACATCCCTGGGGTCAGTGAGAG-3′
G6PD	5′-TGCCCCCGACCGTCTAC-3′	5′-ATGCGGTTCCAGCCTATCTG-3′
FASN	5′-AACTCCAAGGACACAGTCACCAT-3′	5′-CAGCTGCTCCACGAACTCAA-3′
DGAT1	5′-GGTCCCCAATCACCTCATCTG-3′	5′-TGCACAGGGATGTTCCAGTTC-3′
GPAT2	5′-TGACGAAAGCTGATATGCAA -3′	5′-GAGCAGGAGAAACTCCATTT-3′
HMGCS1	5′-CTCTTGGGATGGACGGTATGC-3′	5′-GCTCCAACTCCACCTGTAGG-3′
HMGCR	5′-TAACTCCTCCTTACTCGATAC-3′	5′-AATAGATACACCACGCTCAT-3′
ACADM	5′-ACAACGTGAACCAGGATTAG-3′	5′-TGGCAAATTTACGAGCAGTA-3′
ACADL	5′-GATTAAAAGCCCAGGATACCGC-3′	5′-AGGTGAGCAACTGTTTTGCCA-3′
CPT1C	5′-ATGGGAATGCGCCCCTTATG-3′	5′-AGGTGGCGGATGTAGTCTTTT-3′
GAPDH	5′-GGATTTGGTCGTATTGGG-3′	5′-GGAAGATGGTGATGGGATT-3′
mtDNA tRNALeu	5′-CACCCAAGAACAGGGTTTGT-3′	5′-TGGCCATGGGTATGTTGTTA-3′
nuDNA β2-microglobulin	5′- TGCTGTCTCCATGTTTGATGTATCT-3′	5′- TCTCTGCTCCCCACCTCTAAGT-3′
telDNA	5′- GGTTTTTGAGGGTGAGGGTGAGGGTGAGGGTGAGGGT-3′	5′- TCCCGACTATCCCTATCCCTATCCCTATCCCTATCCCTA-3′
36B4	5′- CAGCAAGTGGGAAGGTGTAATCC-3′	5′- CCCATTCTATCATCAACGGGTACAA-3′
TP53	5′- TCAACAAGATGTTTTGCCAACTG-3'	5′- ATGTGCTGTGACTGCTTGTAGATG-3'

To measure the expression of TP53 and the PGC-related genes, total RNA was extracted from ~1.5 × 10^6^ CD4 T cells following 48 h KML001 treatment or TP53 knockdown (KD) using the PureLink RNA Mini Kit (Invitrogen), and cDNA was synthesized using High Capacity cDNA Reverse Transcription Kit (Applied Biosystems, Foster City, CA) according to the manufacturer’s instructions. RT-qPCR was performed in triplicate. The PCR primer sequences are shown in Table 1. The cycling conditions were as follows: 1 cycle at 95 °C for 3 min, followed by 40 cycles at 95 °C for 10 s and 60 °C for 30 s. Gene expression was calculated using the 2^−ΔΔct^ method, normalized to GAPDH levels, and is presented as fold changes.

To assay 8-oxoGuanine (8-oxoG) lesions on telomeric DNA, genomic DNA was purified from CD4 T cells using the PureLink genomic DNA kit (Thermo Fisher Scientific). DNA concentration was determined using the Synergy H1 BioTek plate reader. Genomic DNA (10 μl, 40 ng/μl) was treated with 1.85 μl RNase free water, 1.5 μl 10× NE buffer, 0.15 μl 100× BSA, and 1.5 μl human 8-oxoGuanine glycosylase (Fpg, New England Biolabs, Ipswich, MA) (treatment mixture) or 3.35 μl RNase free water, 1.5 μl 10× NE buffer, and 0.15 μl 100× BSA (non-treatment mixture) for a total reaction volume of 15 μl. Mixtures were incubated at 37 °C for 16 h, followed by 15 min at 60 °C to inactivate the enzyme. DNA was collected and a fragment (35 ng) of telomeric DNA was amplified using qPCR. The PCR primers are shown in Table 1. For telomere expression, 270 nM and 900 nM of forward and reverse final primer concentrations were used. The cycling conditions for telomeric DNA amplification were as follows: 1 cycle at 95 °C incubation for 10 min (to activate the polymerase), followed by 18 cycles at 95 °C for 15 s and 54 °C for 2 min. The qPCR protocol for 36B4 was as follows: 95 °C incubation for 10 min (to activate the polymerase), followed by 30 cycles at 95 °C for 15 s and 58 °C for 1 min. For 36B4 expression, 300 nM and 500 nM of forward and reverse final primer concentrations were used, respectively^31^. The telomeric DNA content was determined using the following equations: a. ΔCq = (36B4 Cq − telDNA Cq). b. Relative telomeric DNA content = 2 × 2^ΔCq^.

### Western blotting

Purified CD4 T cells were treated with 5 μM KML001 or DPBS control for different times and nucleofected cells were harvested and lysed on ice in RIPA lysis buffer (Boston BioProducts Inc, Ashland, MA) in the presence of protease inhibitors (Thermo Fisher Scientific). The protein concentrations were measured by the Pierce BCA protein assay kit (Thermo Fisher Scientific). The proteins were separated by SDS-PAGE and transferred to polyvinylidene difluoride membranes. The membranes were blocked with 5% non-fat milk, 0.1% Tween-20 in Tris-buffered saline (TBS), and then incubated overnight with primary antibodies for Fasn, PGC1α, NRF1, Pepck, G-6-PD, hmgcs1, ERRα, GPAT, mtTFA, GPx, SOD1, γH2AX, PARP1, TRF2, β-Actin (Cell Signaling, Danvers, MA), PGC1β, HMGCR, DGAT1, PPARα, ACADL, ACADM (Abcam), p53 (Santa Cruz Biotechnology, Dallas, TX), and NEIL1, XRCC1 (Novus Biologicals). Appropriate horseradish peroxide-conjugated secondary antibodies (Cell Signaling) were then used, and the proteins were visualized with the Amersham ECL Prime Western Blotting Detection Reagent (GE Healthcare Bio-Sciences, Pittsburgh, PA). The protein bands were captured and quantitatively analyzed by Chemi DocTM MP Imaging System (Bio-Rad).

### Flow-FISH and meta-FISH analysis of telomere length

CD4 T cells were cultured in RPMI-1640 medium containing 10% FBS (Atlanta Biologicals, Flowery Branch, GA), 100 IU/ml penicillin, and 2 mM L-glutamine (Thermo Scientific), along with 5 µM of KML001 (Sigma-Aldrich) or DPBS as a control at 37 °C and 5% CO_2_ atmosphere for 72 h. Flow-FISH analysis for telomere length was carried out as described previously^21^. For meta-FISH analysis, cells were also cultured in the presence of 1 μg/ml anti-CD3/CD28 antibodies. KML001-exposed cells were treated with 0.1 µg/ml colcemid for 2 h and harvested by centrifugation. The cells were then incubated in 75 mM KCl hypotonic buffer at 37 °C for 10 min or 20 min at room temperature, followed by fixation in methanol and glacial acid acetic (3:1). The cells were mounted on microscopic slides and dried overnight before fixation in 4% formaldehyde. The slides were treated with RNaseA and Pepsin at 37 °C and then dehydrated in successive ethanol solutions of 70%, 90%, and 100%. Telomere FISH was conducted using a FITC-488 conjugated telomeric PNA probe (TelC-FITC488 (CCCTAA)_3_) (PNA Bio, Newbury Park, CA; Cat #F1004) at 37 °C for 4 h, and the slides were then washed in hybridization buffer and mounted overnight at room temperature in Diamond AntiFade with DAPI (Invitrogen). Approximately 50 cells in metaphase in each treatment were counted by Leica SP8 confocal microscope, and the percentages (%) of telomere ends without visible telomere FISH signal were calculated.

### Southern blotting

Southern blot of telomere terminal restriction fragments (TRF) was done as previously described^32^. Briefly, after 5 day KML001 treatment of the cells, DNA was extracted and digested with *HinfI* and *RsaI* restriction enzymes to remove nontelemetric DNA. DNA fragments were separated on 0.5% agarose gel according to their sizes, blotted, detected by a DIG-labeled (CCCTAA)_3_ probe, and visualized by chemiluminescence.

### Confocal microscopy

The CD4 T cells were harvested after treatment with 5 µM KML001 or DPBS control for 6 or 48 h, fixed in 2% PFA for 20 min, followed by permeabilization with 0.3% Triton X-100 in PBS for 10 min. The cells were blocked with 5% BSA in PBS for 1 h and incubated with rabbit anti-OGG1 antibody and mouse anti-TRF1 antibody (Novus Biologicals) at 4 °C overnight. The cells were washed with PBS containing 0.1% Tween-20 three times and stained with anti-rabbit IgG-Alexa Fluor 488 and anti-mouse IgG- Alexa Fluor 555 (Thermo Scientific) at room temperature for 1 h, washed, and mounted with DAPI Fluoromount-G (SouthernBiotech, Birmingham, AL). Images were acquired with a confocal laser-scanning inverted microscope (Leica Confocal, Model TCS SP8, Germany).

### TP53 and TRF2 Knockdown

CD4 T cells were purified from PBMCs isolated from HS and stimulated with 2 µg/ml anti-CD3 and 4 µg/ml anti-CD28 for 3 days in 10% FBS cRPMI with 30 U/ml IL-2. TP53 or TRF2 crRNP was formed following a previously published protocol^33^ and used to transfect stimulated CD4 T cells using the Lonza P3 Primary Cell 4D X Kit L and program EH115, following the manufacturer’s instructions (Lonza, Basel, Switzerland). For the siRNA KD, 100 nM P53 siRNA (Dharmacon, Lafayette, CO) was used for each nucleofection with the program EO115. The cells were harvested at day 3 after nucleofection for western blotting, PCR, apoptosis, MG, Seahorse, ATP, and mtDNA/nuDNA analyses.

### Statistical analysis

Data were analyzed using Prism 7 software and are expressed as mean ± SEM. Outliers were identified by the ROUT method (Q = 1.000%) and excluded from the analysis. Comparisons between two groups were made using a parametric paired or unpaired t-test (with or without Welch’s correction for unequal or equal SDs, respectively) for normally distributed data or non-parametric Wilcoxon paired *t*-test or Mann Whitney *U*-test for non-normal distributions. *P*-values of <0.05 or <0.01 were considered statistically significant or very significant, respectively.

## Results

### Telomere damage by KML001 impairs mitochondrial functions in human CD4 T cells

KML001 is an arsenic compound that directly binds to telomeric sequences, causing telomeric DNA damage and telomere erosion^19,20^. We have recently shown that human T cells treated with KML001 exhibit a senescent state with shortened telomeres^21^. Since mitochondrial dysfunction is another feature of senescent cells, it prompted us to investigate whether telomere injury impacts mitochondrial biology in human T cells. We thus employed this specific telomere-targeting drug as a tool. By culturing CD4 T cells isolated from HS in the presence or absence of KML001 for different time points, we were able to assess mitochondrial functions by measuring MG, MO, mitochondrial DNA relative to nuclear DNA (mtDNA/nuDNA) content, oxygen consumption rate (OCR), extracellular acidification rate (ECAR), ATP production, and ROS.

**Fig. 1:**
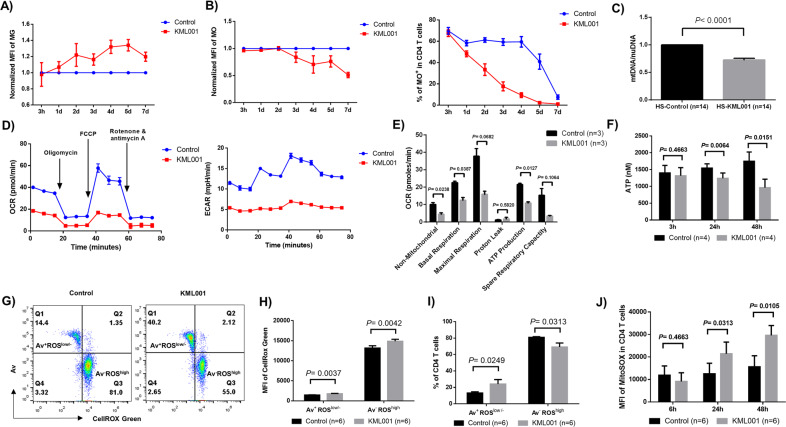
Telomere damage by KML001 impairs mitochondrial functions in human CD4 T cells. Human CD4 T cells were purified from peripheral blood mononuclear cells (PBMCs) by magnetic beads and cultured with or without 5 μM of KML001 for the indicated times, followed by measuring: **A** MitoTracker Green (MG) and **B** MitoTracker Orange (MO) by flow cytometry. Mean fluorescent intensity (MFI) and/or percentage (%) of positive cells are shown (*n* = 4 per group). **C** Purified CD4 T cells were treated with or without 5 μM KML001 for 48 h. The genomic DNA was isolated and mitochondrial DNA (mtDNA) relative to nuclear DNA (nuDNA) content was determined by qPCR (*n* = 14). **D**, **E** The CD4 T cell oxygen consumption rate (OCR) and extracellular acidification rate (ECAR) were measured by Seahorse XFp Cell Mito Stress Tests. Basal respiration, maximal respiration, spare respiratory capacity, and ATP production in KML001 and control (DPBS)-treated cells are shown (*n* = 3). **F** ATP production was measured by a luminescence test in CD4 T cells treated with or without KML001 for the indicated times (*n* = 4). **G–I** Representative dot plots and summary data of the MFI and percentages of CellRox Green (total ROS), Av^+^ ROS^low^, and Av^-^ ROS^high^ cells in KML001 and control (DPBS)-treated cells (*n* = 6). All summary data are shown as mean ± SEM. **J** MFI of MitoSOX in CD4 T cells treated with or without KML001 for the indicated times (*n* = 6).

MG is a green-fluorescent stain that appears to localize in mitochondria regardless of mitochondrial membrane potential. MG is commonly used as a marker for mitochondrial mass since it selectively binds to free thiol groups of cysteine residues enriched in mitochondrial proteins. MG increases as mitochondria swell, which commonly occurs in senescent T cells and is characterized by an accumulation of giant non-functional mitochondria^34^. Indeed, as shown in Fig. 1A, following exposure to KML001, CD4 T cells showed an increased mean fluorescence intensity (MFI) of MG from day 1 to day 7 compared to control, indicating an increased mitochondrial mass or mitochondrial swelling in aging T cells induced by the KML001 treatment.

MO is an orange-fluorescent dye that stains mitochondria in live cells and its accumulation is dependent upon the membrane potential. It is oxidized and therefore retained in actively respiring mitochondria, allowing assessment of the mitochondrial membrane potential and oxidative phosphorylation. As shown in Fig. 1B, human CD4 T cells exposed to KML001 showed a time-dependent decrease in the MFI (left) and the percentage (right) of MO positive cells compared to control.

In addition to MG and MO, we measured mtDNA versus nuDNA content by real-time qPCR. As shown in Fig. 1C, CD4 T cells treated with KML001 for 48 h exhibited a significant decrease in mtDNA copy number relative to nuDNA content compared to cells treated with DPBS control, indicating that telomere injury by KML001 either inhibits mtDNA replication and recombination or increases mtDNA degradation and mitophagy.

We also determined whether telomere injury by KML001 can suppress the mitochondrial respiratory chain in human T cells by Seahorse XFp Cell Mito Stress Tests for OCR as well as ECAR at basal conditions and after sequential injection of oligomycin, FCCP, and rotenone/antimycin A, which is an ideal approach to kinetically assess mitochondrial oxygen consumption, proton flux, glycolysis, and energy metabolism (Fig. 1D). Indeed, T cell mitochondrial respiration, including basal and maximal respirations, and spare respiratory capacities, were inhibited by the 48 h KML001 treatment (reduced by 44.4%, 58.1%, and 78.1%, respectively) (Fig. 1E). This indicates poor mitochondrial fitness and flexibility of T cells exposed to KML001. Consistent with the low mitochondrial membrane potential as evidenced by decreased MO (Fig. 1B), KML001-treated T cells also exhibited increased proton leak and significantly decreased ATP generation rates (Fig. 1E). Given the critical role of mitochondria as the energy powerhouse in cellular activities, we also measured T cell ATP production by a luminescent assay. As shown in Fig. 1F, after treatment with KML001 for 24 or 48 h, CD4 T cells displayed a significantly reduced capacity of ATP generation. Similar results were observed in TCR-stimulated T cells with KML001 treatment at these time points (data not shown).

ROS, which are primarily generated by mitochondria, serve as a faithful readout of cell oxidation. Exposure to KML001 has been associated with the generation of ROS in cancer cells, causing chromosome instability and aberration^35^, and this is considered as a potential mechanism for arsenic-induced telomere genotoxicity. To determine whether KML001 can induce toxic ROS, causing telomere-mitochondrial damage and cell apoptosis, CD4 T cells were isolated from HS and cultured with or without KML001 for 48 h. Because cell apoptosis can affect the accuracy of ROS measurement, we employed a fluorescence-based method (CellROX Green) to measure ROS production and assessed its relationship with cell apoptosis. Fig. 1G shows representative dot plots of cells that were gated into two major populations: Av^+^ ROS^low^ and Av^-^ ROS^high^. Notably, in both KML001 and control (DPBS) treated T cells, apoptotic cells (Av^+^) produced less ROS (ROS^low^) and live cells (Av^-^) produced more ROS (ROS^high^). Interestingly, the frequency of Av^+^ ROS^low^ cells increased, but the Av^-^ROS^high^ cells decreased upon KML001 treatment. Summary data show the MFI of both Av^+^ ROS^low^ and Av^-^ ROS^high^ subsets increased in KML001-treated cells compared to the control (Fig. 1H). However, while the percentage (%) of Av^+^ ROS^low^ cells increased, the frequency of Av^-^ ROS^high^ cells decreased in KML001-treated cells compared to the control (Fig. 1I). These data suggest that the intracellular ROS generated by CD4 T cells with KML001 treatment may play an important role in triggering cell apoptosis and that the apoptotic, dying cells produce less ROS. In addition to measuring total cellular ROS, we further examined mitochondrial ROS by MitoSOX after treating CD4 T cells with KML001 for 6, 24, or 48 h. As shown in Fig. 1J, we observed a gradual increase in the MFI of MitoSOX following prolonged exposure to KML001. Notably, KML001-treated cells showed a significantly increased MFI of MitoSOX after 24 and 48 h, but not at the early 6 h time point, compared to the control. Taken together, these results strongly suggest that telomere targeting by KML001 is associated with and could be a key factor per se in mitochondrial dysfunction, as evidenced by the aberrant mitochondrial density, compromised mitochondrial membrane potential and oxidative phosphorylation, decreased oxygen consumption, glycolysis metabolism, and ATP generation, and increased oxidative stress in human T cells.

### Telomere injury by KML001 represses the PGC-1α network governing mitochondrial biogenesis in T cells

**Fig. 2:**
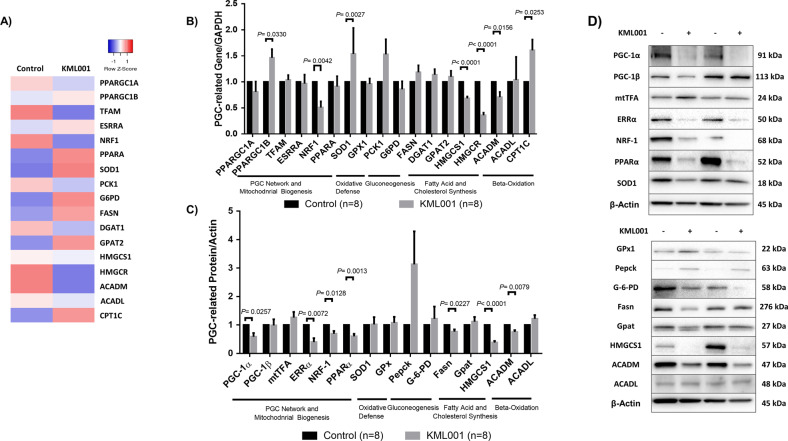
KML001 represses the PGC-1α network that governs mitochondrial biogenesis in human T cells. **A** Heatmap representing the expression of the PGC network and target genes involved in governing metabolism (*n* = 2). **B** RT-qPCR analysis of PGC-1α network genes governing mitochondrial biogenesis, oxidative defense, gluconeogenesis, fatty acid, and cholesterol synthesis as well as beta-oxidation in CD4 T cells treated with KML001 or DPBS control for 48 h (*n* = 8). **C**, **D** Summary data (middle lower panel, C) and representative images (right, D) of western blots of PGC-1-related genes regulating mitochondrial metabolic activities in CD4 T cells treated with KML001 or DPBS for 48 h (n = 8). All summary data are shown as mean ± SEM.

How telomere injury by KML001 induces mitochondrial dysfunction remains unclear. Recent studies in mice revealed that PGC-1α and PGC-1β, master regulators of mitochondrial biogenesis and metabolism, were suppressed across aging tissues^24,25^. It is unknown whether PGCs are inhibited and play a role in KML001-induced mitochondrial dysfunction in human T cells. To uncover the influence of KML001-induced telomere damage on mitochondrial dysfunction, we performed gene array analysis to examine the expression of genes governing cellular metabolism in CD4 T cells treated with KML001 or DPBS control. Table 2 highlights the up- and downregulated genes with fold changes >2 (averaged between two HS) following KML001 treatment for 48 h. Given the critical role of the PGC network in regulating cellular metabolism, we also investigated the transcriptional profile of the PGC network and their target genes involved in governing mitochondrial biogenesis and oxidative phosphorylation (ESRRA, NRF-1, PPARA, TFAM), oxidative defense (SOD1, GPX1), gluconeogenesis (PCK1, G6PD), fatty acid and cholesterol synthesis (FASN, DGAT, GPAT, HMGCS1, HMGCR), and β-oxidation (ACADM, ACADL, CPT1C). Figure 2A shows the heatmap of these gene expressions in the PGC network (averaged between two HS).

**Table 2:** Up and downregulated metabolic genes in CD4 T cells with >2 fold changes.

Category	Gene	Fold change
OXPHOS	SDHB	−2.72317665
	BAX	2.25509595
	IDH2	−3.3836822
	UQCFRS1	−2.3892804
	NDUFA3	−2.0965658
	COX11	−2.16494835
	NDUFB4	−6.04910535
Glycolysis	PFKFB2	2.30761945
	PFKFB4	−2.06727665
Lactate production and transporters	SLC16A4	−2.50145315
	SLC16A10	−2.0563729
Gluconeogenesis	GPT	2.28088635
	GPT2	2.1976123
	ADH5	−2.47808485
	GALM	−2.0332245
Glycogen metabolism	GYS2	−2.582366
Hexosamine metabolism	GNPNAT1	−2.84449395
Pentose phosphate pathway	RPIA	−2.2417299
	TALDO1	2.4924492
Glycerol/fatty acid/cholesterol synthesis	ACACB	2.3214025
	ACAT1	−2.0035285
	ACSM3	−2.887862
	GPD1L	−2.20293675
Serine/ glycine/one-carbon metabolism	DNMT3B	−2.56062905
	GCSH	−2.8607831
	GLDC	2.34707975
	MAT2B	−2.0632765
	MTHFD2	−2.7761227
	PSAT1	2.5371184
TCA cycle	FH	−2.2149622
	PDK1	−4.4372955
	SDHAF1	2.2466182
	SUCLG2	−2.4647895
Glutamine transporters and glutaminolysis	SLC1A5	2.5634322
Redox balance	GCLM	7.02115825
	ME2	−3.1165164
Fatty acid oxidation	ACADSB	−4.23406895
	ALDH3A2	−2.1076536
	HADH	−4.00500305
	PAFAH1B2	−2.0055203
	ACSL4	−3.1240279
Nucleotide metabolism	ADCY4	5.6766289
	AK5	−4.1834393
	AMPD3	3.6734286
	DCK	−2.4675163
	PAICS	−2.0157439
	PNP	−3.19877885
	PPAT	−2.25211545
	RRM1	−2.4361025
	RRM2	−2.15779715
	TK1	−2.1033255
	TYMS	−7.74290015
	UPP1	−4.65474695
Mitochondrial biogenesis	TFAM	−2.80362125

Based on these results, we further analyzed the genes of the PGC network by RT-qPCR in CD4 T cells treated with KML001 or DPBS control for 48 h. Among the genes examined, NRF-1, HMGCS1, HMGCR, and ACADM were significantly downregulated, whereas PPARGC1B, SOD1, and CPT1C were remarkably upregulated by the KML001 treatment (Fig. 2B). Since mRNA levels may not necessarily correlate linearly with protein levels, we also examined the protein levels of these genes by western blotting. As shown in Fig. 2C, D (summarized data and representative imaging), PGC-1α, ERRα, NRF-1, PPARα, Fasn, HMGCS1, and ACADM protein levels were significantly suppressed in CD4 T cells treated with KML001 for 48 h.

**Fig. 3:**
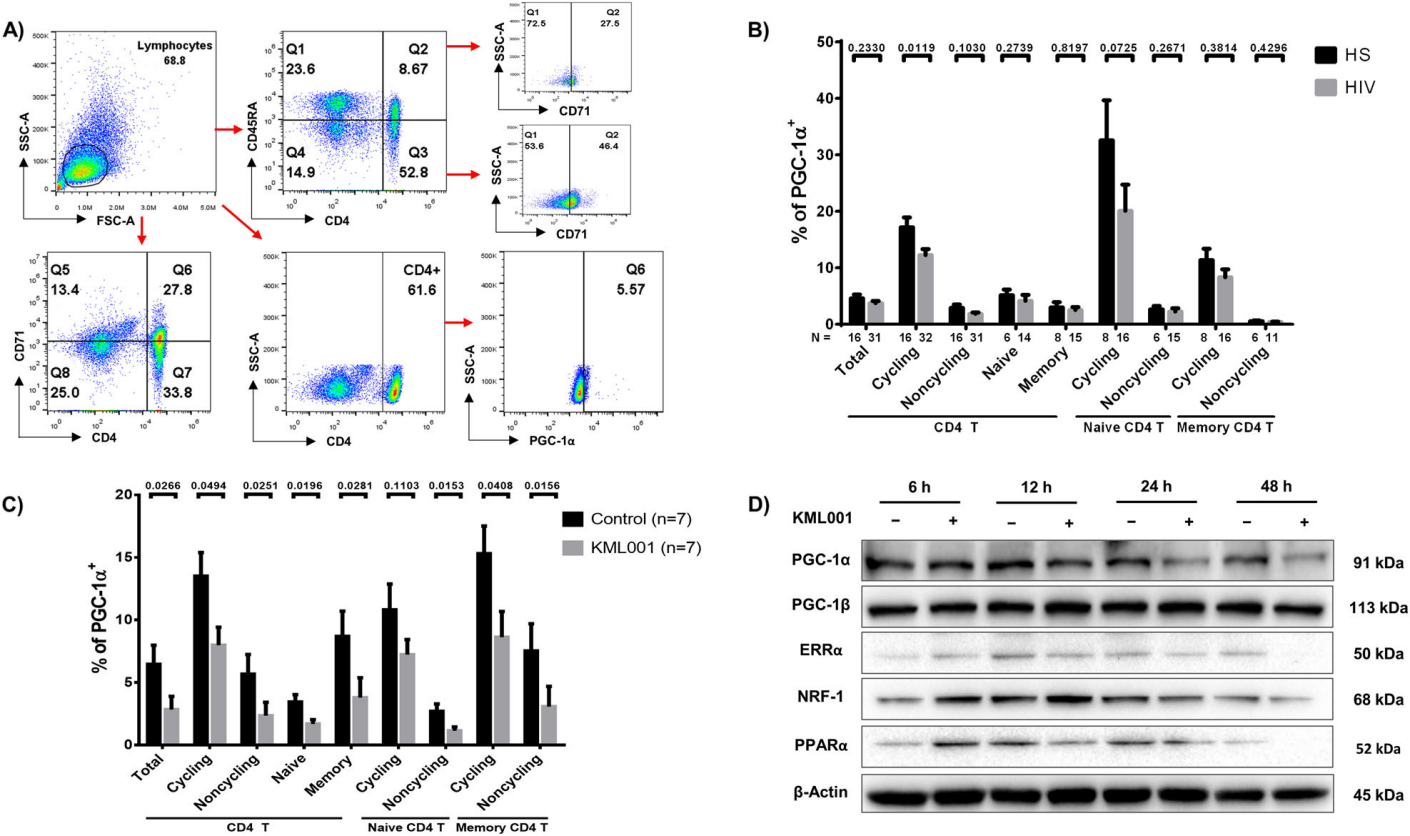
KML001 disrupts CD4 T cell homeostasis and represses key PGC-1α targets in a time-dependent manner. **A** Representative dot plots for flow cytometry gating strategy for lymphocytes, naive and memory, cycling and non-cycling, CD4, and PGC-1α in PBMCS from PLHIV or HS. **B**, **C** Flow cytometry analysis of the frequencies (%) of PGC-1α^+^ cells in total, cycling (CD71^+^), non-cycling (CD71^−^), naive (CD45RA^+^), and memory (CD45RA^−^), cycling naive (CD71^+^ CD45RA^+^), non-cycling naive (CD71^−^ CD45RA^+^), and cycling memory (CD71^+^ CD45RA^−^), and non-cycling memory (CD71^−^ CD45RA^-^) CD4 T cell subsets in PBMCs isolated from people living with HIV (PLHIV) versus healthy subjects (HS) (**B**) or in CD4 T cells after treatment with KML001 or DPBS for 48 h (*n* = 7) (**C**). **D** Representative images of western blot analysis of PGC-1α, PGC-1β, ERRα, and NRF-1, and PPARα expressions in CD4 T cells treated with KML001 or DPBS for 6, 12, 24, and 48 h. All summary data are shown as mean ± SEM.

Given the known role of telomeric DNA damage in T cell dysfunction during chronic viral infections^14,15,21,36–38^, and that only PGC-1α, but not PGC1β, was inhibited by KML001 treatment (Fig. 2C, D), we examined the frequencies of PGC-1α^+^ cells in CD4 T cell subsets in PLHIV, in which we have recently shown an aging phenotype with shortened telomeres^36^ and compromised mitochondria (unpublished observations). PBMCs were isolated from PLHIV and HS, followed by staining of total (CD4^+^), cycling (CD71^+^), non-cycling (CD71^−^), naive (CD45RA^+^), memory (CD45RA^−^), cycling naive (CD71^+^CD45RA^+^), non-cycling naive (CD71^−^CD45RA^+^), cycling memory (CD71^+^CD45RA^−^), and non-cycling memory (CD71^−^CD45RA^-^) CD4 T cells. Figure 3A shows representative dot plots for the gating strategy to determine the frequency of PGC-1α^+^ cells in each subset of CD4 T cell populations. Lymphocytes were first gated, followed by CD4^+^ cells, CD45RA^+/−^ naive/memory cells, CD71^+/−^ cycling/non-cycling cells, and then PGC-1α^+^ cells in these populations. As shown in Fig. 3B, the frequencies of PGC-1α^+^ cells were reduced in all cell subsets in PLHIV but only significantly in cycling CD4 cells, highlighting the potential connection between telomeric DNA damage and deregulation of the PGC-1α network, and thus mitochondrial dysfunctions. We then examined the expression of PGC-1α following 48 h KML001 treatment in purified CD4 T cells by flow cytometry. As shown in Fig. 3C, the frequencies of PGC-1α^+^ cells were significantly repressed by the KML001 treatment in all CD4 T cell subsets. These results suggest that KML001-induced mitochondrial dysfunction may be regulated by the disruption of the PGC-1α family of transcription coactivators in CD4 T cells, which controls many aspects of mitochondrial biology and cellular metabolism^39^. In order to identify key molecules that may be influenced by this PGC-1α deregulation, we also measured the MFI of NRF-1 in CD4 T cell subsets by flow cytometry following 48 h exposure to KML001. Similar to PGC-1α, the MFI of NRF-1 was reduced in all cell subsets following KML001 treatment (data not shown). Given the critical role of the PGC-1 network in mitochondrial dysregulation, we further treated CD4 T cells with KML001 or control for 6, 12, 24, or 48 h and examined the protein expressions of PGC-1α, PGC-1β, ERRα, NRF-1, and PPARα by western blotting. As shown in Fig. 3D, the expressions of PGC-1α, ERRα, NRF-1, and PPARα, but not PGC-1β, in CD4 T cells were suppressed at 12 or 24 h, and 48 h following KML001 treatment. Collectively, these data indicate the deregulation of the mitochondrial PGC-1α network following exposure to the telomere-targeting drug KML001.

### DNA base excision repair machineries are impaired in CD4 T cells exposed to KML001

**Fig. 4:**
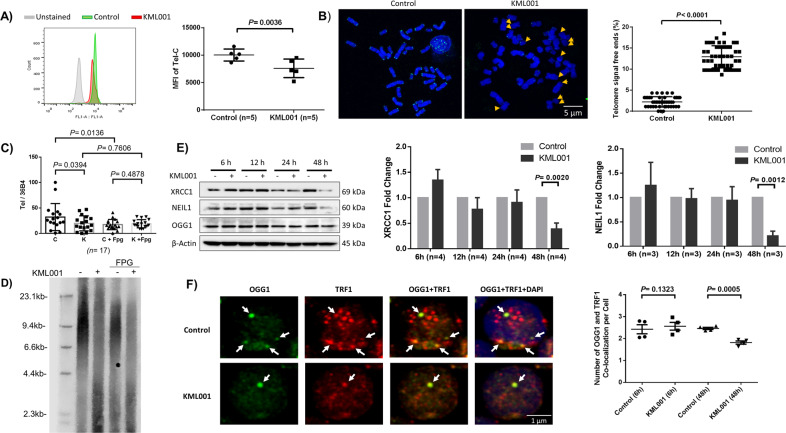
CD4 T cells treated with KML001 exhibit telomere erosion, disruption of the BER pathway, and p53 activation. **A** Telomere length in CD4 T cells exposed to KML001 or control for 72 h was measured by flow-FISH (*n* = 5). **B** Representative images and summary data of the percentage (%) of chromosomes with telomere free ends in CD4 T cells treated with KML001 or DPBS in the presence of anti-CD3/CD28 antibody stimulation for 72 h, and then analyzed by meta-FISH. Approximately 50 cells in metaphase were counted using confocal microscopy (scale bar is 5 μm). **C** Summary data for qPCR of telomeric DNA content following exposure of CD4 T cells to KML001 for 5 days and DNA glycosylase Fpg (*n* = 17). **D** Representative image of the Southern blot of telomeric DNA following exposure of CD4 T cells to KML001 for 5 days and DNA glycosylase Fpg. **E** Representative images and summary data of western blot analysis of the BER-related XRCC1 and NEIL1 expressions in CD4 T cells treated with KML001 or DPBS control for 6, 12, 24, and 48 h (XRCC1, *n* = 4; NEIL1, *n* = 3). **F** Confocal microscopy representative images and summary data (*n* = 4) of the co-localization of OGG1 with TRF1 in CD4 T cells treated with KML001 or DPBS control for 48 h (scale bar is 1 μm). All summary data are shown as mean ± SEM.

We next investigated how telomeric DNA is damaged and remains unrepaired to compromise mitochondrial functions in human T cells exposed to KML001. Telomeres are nucleoprotein complexes at chromosome ends that function to preserve chromosomal integrity and cell viability^9–12^. As shown in Fig. 4, CD4 T cells exposed to KML001 for 72 h exhibited significantly shortened telomeres, as measured by flow-FISH (Fig. 4A). Telomere shortening and attrition were confirmed by the meta-FISH assay (Fig. 4B). We have recently shown that telomeres in KML001-treated T cells were not only shortened but also DNA damaged, as evidenced by the accumulation of dysfunctional telomere-induced foci (TIFs)^21^. Notably, repeated telomeric sequences (TTAGGG) are enriched in guanine (G), which is particularly sensitive to oxidative stress and is especially vulnerable to ROS, where it forms 8-oxoG lesions. Thus, we measured 8-oxoG accumulation in the telomeric DNA of CD4 T cells exposed to KML001 using qPCR (Fig. 4C) and southern blotting (Fig. 4D). We employed an 8-oxoguanine DNA glycosylase, formamidopyrimidine [fapy]-DNA glycosylase (Fpg), which is capable of functioning as both an N-glycosylase and AP-lyase. This dual activity allows the enzyme to release damaged purines from double-stranded DNA, creating an apurinic AP site that can be cleaved by the AP-lyase activity (both 3′ and 5′ to the AP site), removing the AP site and generating a one base gap. Fpg-mediated digestion of DNA can thus identify and remove 8-oxoG lesions^40–42^. Following 5-day exposure to KML001, telomeric DNA content was significantly reduced. However, while the PCR assays showed increased overall 8-oxoG lesions by the treatment of cellular DNA with Fpg (an 8-oxoguanine DNA glycosylase), there was no difference in telomeric DNA content between KML001 and control following DNA treatment with Fpg. Nevertheless, southern blot analysis of treated DNA revealed shortened telomeres in KML001-exposed cells, especially in KML001-exposed, Fpg-treated DNA (Fig. 4D). These results indicate that telomeres are significantly shortened with DNA damage following KML001 treatment and that 8-oxoG DNA injury may be involved in this process.

We and others have previously shown that chronic viral infections and KML001 treatment can induce ROS overproduction^14,21,36^. Overproduction of ROS leads to oxidative damage in chromosomal DNA, especially on vulnerable telomeres. These DNA single-strand breaks (SSBs) or double-strand breaks (DSBs) are usually repaired by the cellular antioxidant defense system. Specifically, the BER pathway repairs most oxidative lesions and plays an important role in maintaining genomic stability and telomere integrity^43^. One important member of the BER protein family, XRCC1, repairs DNA-SSBs and excises damaged bases caused by ionizing radiation and alkylating agents^44^. Additionally, both NEIL1 and OGG1 are bi-functional glycosylases that recognize oxidative base damage and excise damaged bases by cleaving the DNA backbone at the 3’ end of the abasic site^45^. NEIL1 recognizes other oxidized pyrimidines and hydantoin lesions but not 8-oxoG^41^, and OGG1 specifically recognizes 8-oxoG and is quickly recruited to the telomeres upon 8-oxoG damage on the telomeres^46^. Given the broad telomeric DNA damage we observed, we hypothesized that the KML001-induced ROS production triggers dysfunction of the antioxidant defense system (such as the BER pathways) and consequently results in telomere erosion and cell senescence or death. To test this hypothesis, we performed western blotting to check the expression levels of BER-related proteins, including XRCC1, NEIL1, and OGG1 in CD4 T cells exposed to KML001 for various time-points. As shown in Fig. 4E, the results revealed that KML001 treatment for 48 h dramatically decreased the levels of XRCC1 and NEIL1, but not OGG1 expression, compared to the control. Taken together, these findings suggest the accumulation of telomeric DNA damage, likely through impaired DNA-BER machinery, in T cells exposed to KML001.

Since OGG1 expression levels were unaffected in KML001-treated cells (Fig. 4E), we further investigated OGG1 recruitment onto telomeres by confocal microscopy. We found that the co-localization of OGG1 and TRF1 (Telomeric Repeat Factor 1, a telomere shelterin protein) was significantly decreased in 48 h KML001-treated cells compared to the control (Fig. 4F). These results indicate that while the expression level of OGG1 was not affected, it could not be recruited to the telomeres to remove 8-oxoG sites, leading to telomeric DNA damage and telomere erosion.

### Telomere damage by KML001 directly activates p53, which in turn represses the PGC-1α gene network

**Fig. 5:**
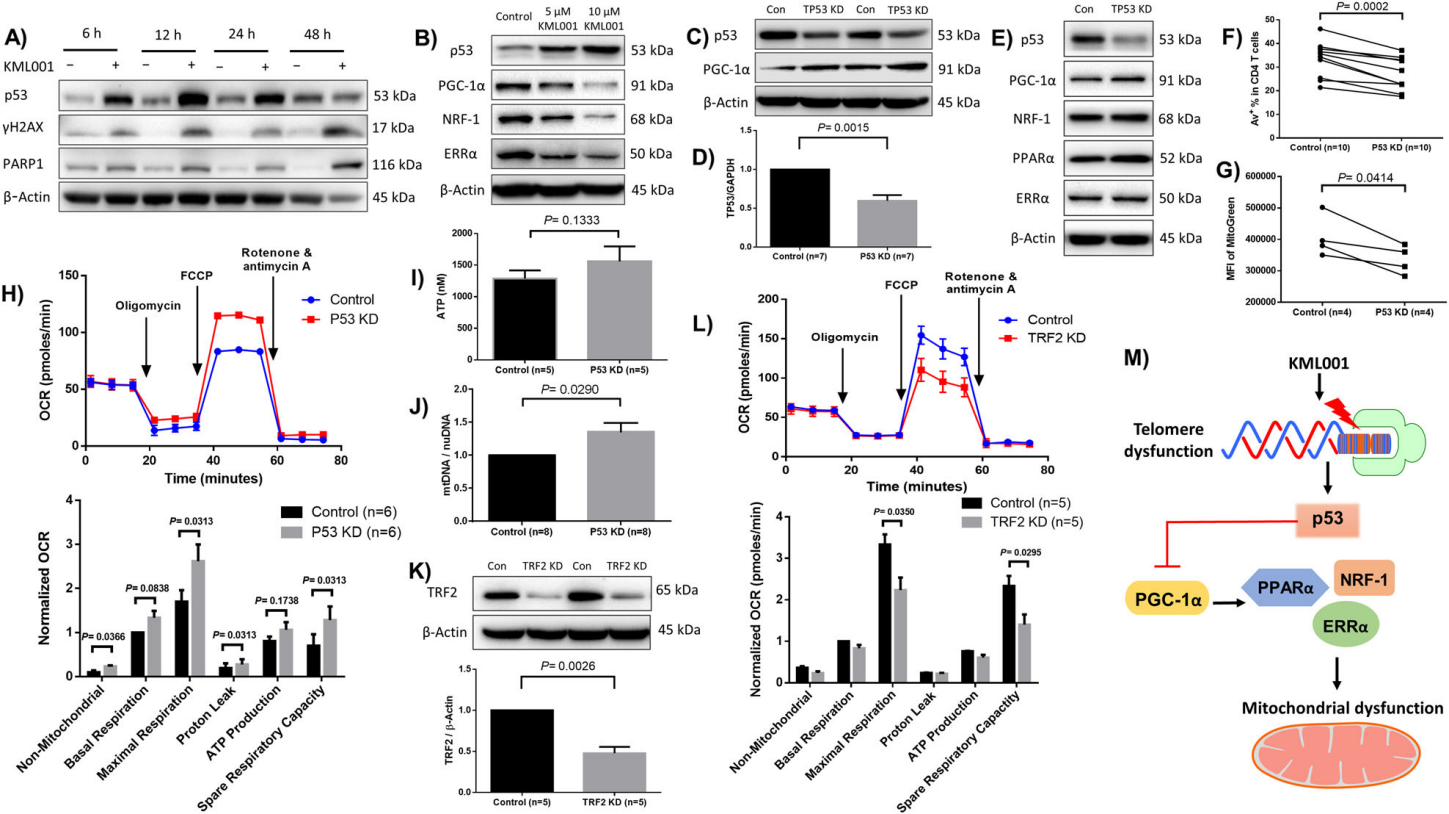
KML001-induced telomeric DNA damage activates p53-mediated repression of the PGC-1α gene network and mitochondrial functions. **A** Kinetic analysis of p53, γH2AX, PARP1 protein levels by western blot in CD4 T cells exposed to KML001 or DPBS control for 6, 12, 24, and 48 h. **B** Representative western blots for the key molecules in the PGC-1α network in CD4 T cells treated with DPBS control, or 5 or 10 μM KML0001 for 48 h in the presence of anti-CD3/CD28 antibody stimulation. **C** Representative western blot bands for the PGC-1α network following TP53 knockdown (KD) by CRISPR/Cas9 nucleofection. **D** Summary data of RT-qPCR analysis of TP53 gene expression following siRNA-mediated TP53 KD (*n* = 7). **E** Representative western blot bands for the PGC-1α network following TP53 KD by siRNA. **F** Summary data for the frequency of apoptotic Av^+^ cells in CD4 T cells following TP53 KD (*n* = 10). **G** Summary data for the MFI of MG in CD4 T cells following TP53 KD (*n* = 4). **H** The CD4 T cell oxygen consumption rate (OCR) was measured by Seahorse XFp Cell Mito Stress Tests, and summary data for basal respiration, maximal respiration, spare respiratory capacity, and ATP production following TP53 KD (*n* = 6) are shown. **I** ATP production measured by a luminescence test in CD4 T cells following p53 KD (*n* = 6). **J** Mitochondrial DNA (mtDNA) relative to nuclear DNA (nuDNA) content was analyzed by qPCR following TP53 KD (*n* = 8). **K** Representative western blot bands and summary data following TRF2 KD (*n* = 5). **L** The oxygen consumption rate (OCR) was measured by Seahorse XFp Cell Mito Stress Tests, and summary data for basal respiration, maximal respiration, spare respiratory capacity, and ATP production following TRF2 KD (*n* = 5) are shown. **M** A model depicting the molecular mechanism linking telomeric DNA damage and mitochondrial compromise via KML001-mediated telomere shortening and p53-mediated repression of the PGC-1α gene network. All summary data are shown as mean ± SEM.

The prevailing view of the mechanism by which telomeres prevent unwanted p53-mediated DDR at chromosomal free ends posits that p53 mediates cellular checkpoints of cell senescence and apoptosis. The relevance of p53 in aging is evidenced by constitutively expressed p53 accelerating early aging phenotypes in mice, whereas p53 deficiency markedly attenuated aging phenotypes^33,47^. We have previously shown that the p53-dependent ubiquitination and proteasomal degradation of telomere-associated shelterin protein, TRF2, causes telomere erosion and premature T cell aging in patients with hepatitis C^15^. Whether p53-mediated DDR is involved in the KML001-mediated mitochondrial dysfunction and T cell senescence or apoptosis remains unknown. To uncover this, we cultured purified CD4 T cells with KML001 for varying time-points, followed by western blot analysis of the protein levels of p53, the DNA damage marker γH2AX, and the apoptosis marker cleaved Poly ADP-ribose polymerase 1 (PARP1)—an enzyme that catalyzes the transfer of ADP-ribose onto target proteins that plays an important role in maintaining DNA damage repair and chromosomal stability^48^. As shown in Fig. 5A, the level of p53 was markedly increased, as early as 6 h after the treatment, along with γH2AX and cleaved PARP1, in CD4 T cells exposed to KML001 over time, indicating an increase in p53-associated DNA damage and cell apoptosis.

To further elucidate the crosstalk between telomere and mitochondrial dysfunctions, we examined the protein expressions of p53, PGC-1α, NRF-1, and ERRα in TCR-stimulated CD4 T cells following treatment with 5 or 10 μM of KML0001 for 48 h. We observed a dose-dependent increase in protein expression for p53 and decreased expression of PGC-1α, NRF-1, and ERRα (Fig. 5B). As expected, p53 increased and PGC-1α/NRF-1/ERRα decreased in a dose-dependent manner, respectively, indicating a potential negative regulation of PGC-1α network proteins by p53. Thus, we employed a novel CRISPR/Cas9 method to knockdown (KD) TP53 in primary CD4 T cells, followed by measuring PGC-1α expression. This method involves delivering the synthesized TP53-crRNA/tracrRNA and Cas9 proteins into TCR-activated (72 h) CD4 T cells by using a primary T cell nucleofection Kit (Amaxa, Lonza)^49,50^. As shown in Fig. 5C, TP53 KD cells showed a significant reduction in p53 expression compared to the control. Notably, the expression of PGC-1α was increased following P53 KD. We also used siRNA for P53-KD, and as expected, RT-qPCR confirmed reduced mRNA expression of TP53 (Fig. 5D), and western blot analysis confirmed increased expression levels of PGC1α, NRF-1, and ERRα following p53 KD (Fig. 5E). Given the critical role of p53 in regulating cell metabolism and survival via checkpoints, we also evaluated cell apoptosis and mitochondrial mass following p53 KD using flow cytometry. As shown in Fig. 5F,G, TP53-KD significantly reduced Av^+^ cell frequencies as well as the MFI of MG, suggesting reduced cell apoptosis and mitochondrial mass in TCR-activated CD4 T cells following p53 KD.

To further assess the outcomes of p53 KD in mitochondrial dysregulation, we measured cell respiration by Seahorse. As shown in Fig. 5H, representative OCR and summary data following p53 KD (*n* = 6), the maximal mitochondrial respirations and spare respiratory capacity were significantly increased in CD4 T cells with p53 KD compared to the control. ATP production was also increased, but not significantly. We further confirmed the increases in ATP levels in TP53 KD cells by a luminescent assay (Fig. 5I). Moreover, TP53 KD led to a significant increase in mtDNA copy numbers relative to the nuDNA content (Fig. 5J).

To further reveal the role of telomere damage in inducing mitochondrial dysfunction, we employed an additional method of generating telomeric DNA damage via TRF2 (a critical telomere shelterin protein) KD (Fig. 5K). Following TRF2 KD, mitochondrial respirations, including maximal respiration and spare respiratory capacity, were significantly compromised (Fig. 5L). We have shown p53 is significantly upregulated following TRF2 KD^15^ or by KML001 treatment, supporting the notion that mitochondrial dysfunction induced by TRF2 KD or telomere-targeting drug is likely mediated by the p53-dependent pathways. Collectively, these results indicate that the p53-PGC-1α-NRF-1 axis regulates mitochondrial functions and T cell fate, providing a link between telomere and mitochondrial injuries.

## Discussion

Telomere erosion and mitochondrial compromise are two major features of aging cells and are observed in T cells during many chronic viral infections^13–18^; however, the mechanisms by which these deregulations occur to alter T cell aging are poorly understood. While these two features have been investigated independently, the possible crosstalk and molecular link between these cellular defects have remained elusive. In the present study, we developed a model system using the telomere-targeting drug KML001 as a tool to investigate the role of telomere injury in inducing mitochondrial dysfunction. We demonstrate that mitochondrial functions are compromised, the PGC-1α network is deregulated, telomeres are shortened with DNA damage, and the BER DNA damage repair pathway is disrupted in CD4 T cells by the KML001 treatment. In addition, we discovered that p53 regulates mitochondrial functions via the p53-PGC-1α pathway.

Tumor suppressor p53 is a key cell cycle regulator. Its role as a DNA damage-sensor is critical for activating DNA damage and repair molecules/pathways to maintain cellular homeostasis and genetic integrity. Upon DNA damage, such as telomere attrition or erosion via persistent cellular over-activation and inflammation, p53 senses DNA damage signals and triggers either DNA damage repair or cell apoptosis. The role of p53 in mediating telomere erosion and DNA damage has been well-established, but herein we show that p53 plays a key role in linking telomeric DNA damage to mitochondrial compromise via the p53- PGC-1α-NRF-1 axis in CD4 T cells following telomere injury induced by KML001 treatment. These results are consistent with a previous study showing significant disruption of the PGC-1α network via the p53 binding-mediated repression of PGC-1α/β in mice null for telomerase RNA component (Terc) or telomerase reverse transcriptase (Tert) genes^24^. This animal model of telomere dysfunction revealed significant deregulation of the PGC network and downstream mitochondrial functions^24^. Here we show that telomeric DNA damage occurs earlier than mitochondrial failure. Our results strongly support a notion that KML001-induced telomeric DNA damage leads to dose- and time-dependent alterations in the expressions of p53, PGC-1α, NRF-1, ERRα, and PPARα proteins, ultimately leading to impaired mitochondrial functions.

Notably, the PGC-1α network has been implicated in a number of clinical conditions, including neurodegeneration, cardiac failure, and cancer, while also being linked to p53 and telomere dysfunction in these disease states^51–53^. Based on published reports and our findings in this study, we propose a model (depicted in Fig. 5M), in which telomeric DNA damage from insults—such as cell over-activation and proliferation, chronic viral infection, cancer, inflammatory cytokines, oxidative stress, aberrant T cell homeostasis, and neurodegenerative and cardiovascular diseases—can activate p53, which in turn represses PGC-1α (a transcriptional coactivator of mitochondrial biogenesis via downstream NRF-1 and ERRα) and ultimately leads to compromised mitochondrial functions.

In summary, our data demonstrate a role for telomeric DNA damage and p53 in the deregulation of mitochondrial activity in CD4 T cells following telomeric DNA insults. Importantly, manipulation of p53 can rescue PGC-1α, NRF-1, ERRα, and PPARα expressions, and restore mitochondrial functions. Therefore, this study establishes an intimate link between telomeric DNA damage and mitochondrial metabolism and provides a novel approach to rejuvenate impaired T cell functions.
